# Supplementary figures and images for: Predicting reliability through structured expert elicitation with the repliCATS (Collaborative Assessments for Trustworthy Science) process
Source: PLoS One. 2023 Jan 26;18(1):e0274429. doi: 10.1371/journal.pone.0274429 (PMC9879480; doi:10.1371/journal.pone.0274429)

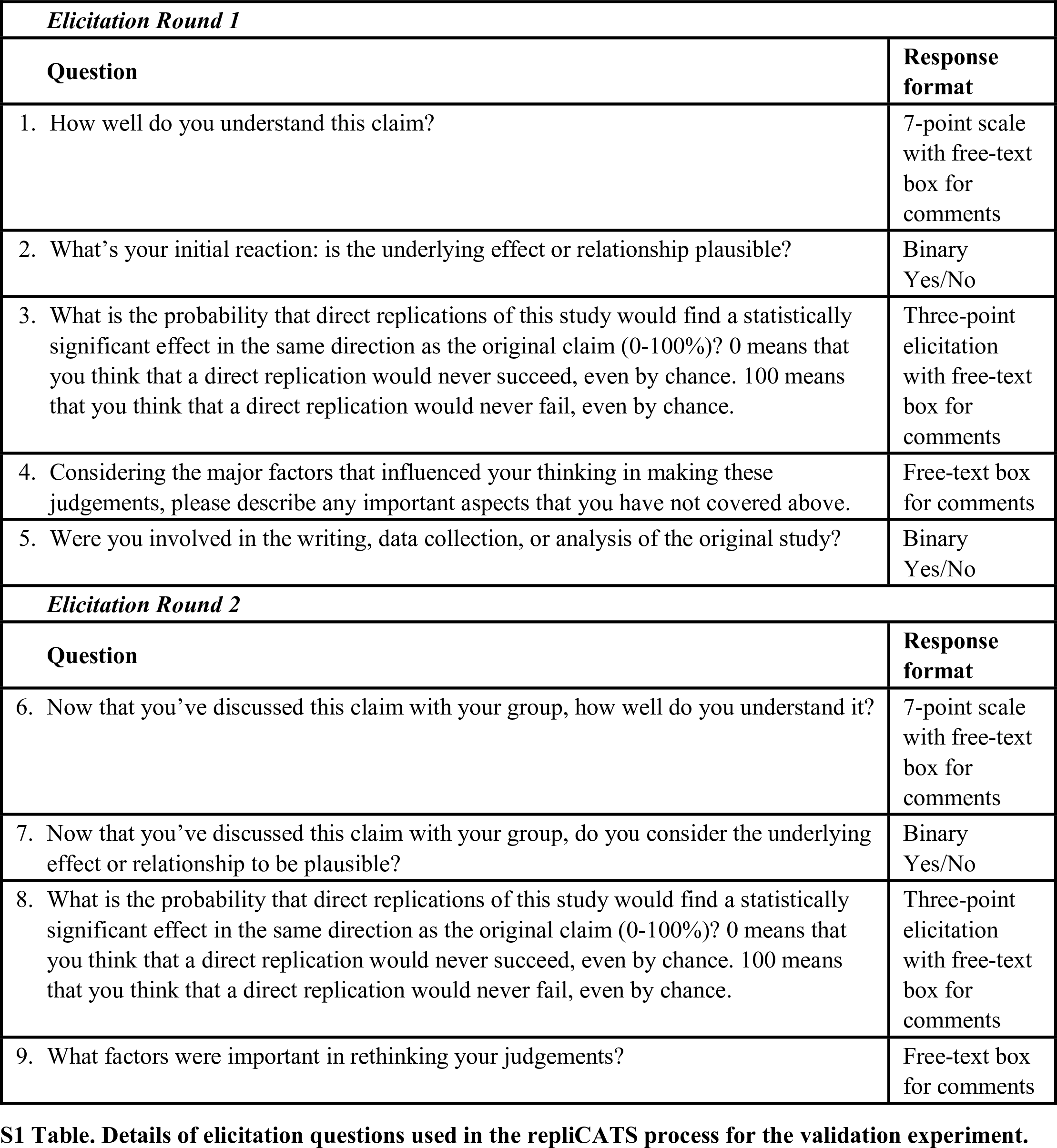

Supplement: S1 Table — (TIF) [file pone.0274429.s001.tif]

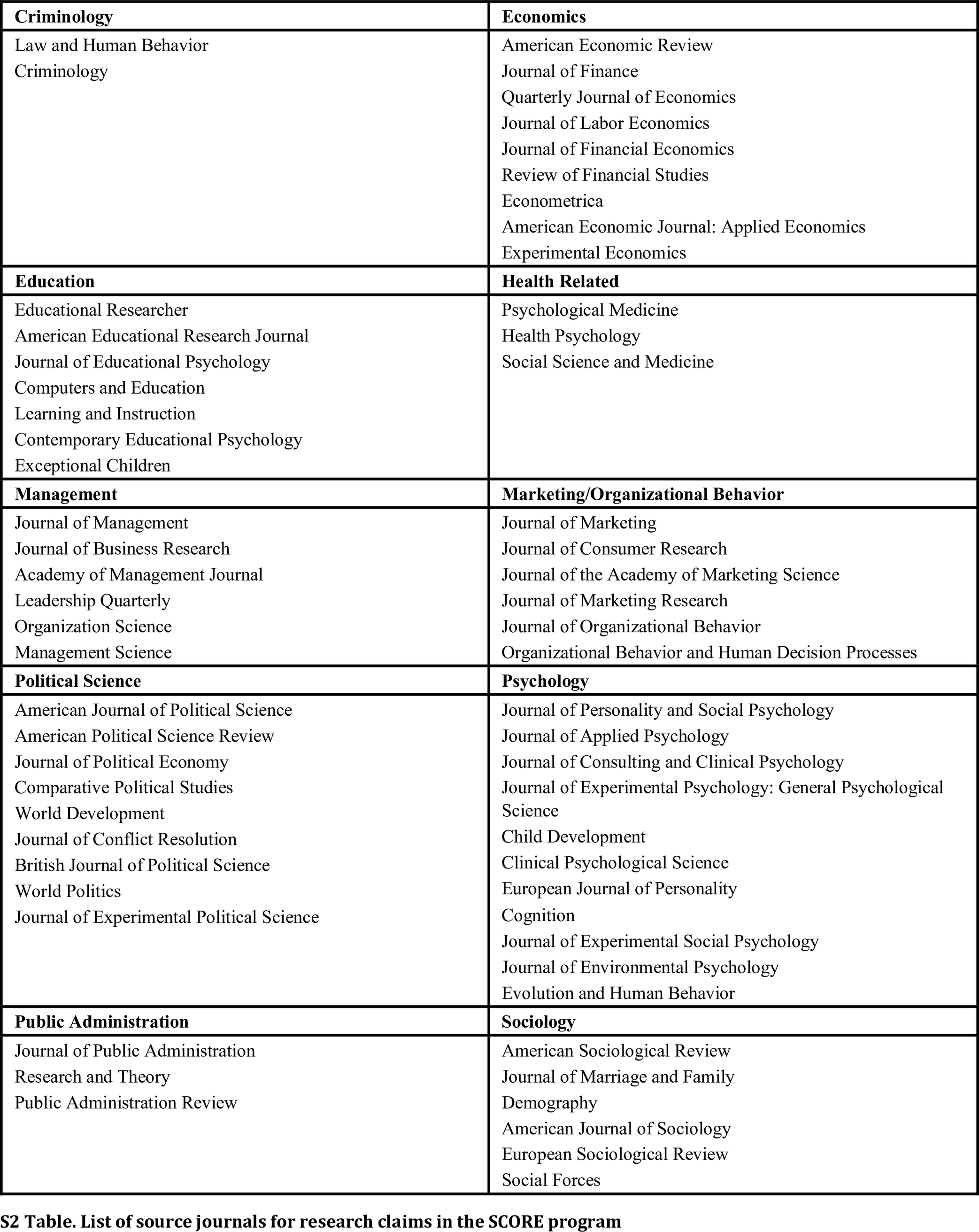

Supplement: S2 Table — (TIF) [file pone.0274429.s002.tif]
