# Supplementary material for: Predicting reliability through structured expert elicitation with the repliCATS (Collaborative Assessments for Trustworthy Science) process
Source: PLoS One. 2023 Jan 26;18(1):e0274429. doi: 10.1371/journal.pone.0274429 (PMC9879480; doi:10.1371/journal.pone.0274429)
Supplement: Attachment — Submitted filename: Responses_Predicting_reliability_repliCATS_20220601.pdf [file pone.0274429.s004.pdf]

Predicting reliability through structured expert elicitation with the repliCATS (Collaborative Assessments for Trustworthy Science) process

Responses to editor

"One requirement is that the tool must meet the criteria of validation, which may be met by including a proof-of-principle experiment or analysis."
We have described in §2.5 more detail regarding the validation experiment and provided a new figure including results. While we regard the results of this as more than a proof-of-concept experiment, we have adjusted the wording of the paper to address this concern, shared by Reviewer 2. The SCORE program has been a large, complex, multi-disciplinary and multi-team project and it has not been possible to cover all of the project's aims, methods and results in a single paper. Moreover, some of the data from the overall program is still embargoed for public release, as the work of some teams is ongoing. However, all data underlying our validation experiment is now publicly available and we have included a link.

"Please include this amended Role of Funder statement in your cover letter; we will change the online submission form on your behalf."
Updated funder statement was provided in the previous cover letter. We will include it in this cover letter.

"Should your manuscript be accepted for publication, we will hold it until you provide the relevant accession numbers or DOIs necessary to access your data."
Data underlying the validation experiment reported in this paper is now publicly available and we have clarified this in re-submission.

"Please upload a new copy of Figure 2 as the detail is not clear."
New Figure 2 was uploaded with previous resubmission, and this new Figure was specifically referred to by Reviewer 3. Could the editor please confirm if further revision is required.

"We note that Figure 2 in your submission contain copyrighted images"
All graphics in Figure 2 were created by the repliCATS project. Could the editor please confirm which graphics are of concern.

"Please provide additional details regarding participant consent."
Accepted; additional details provided in §3 as requested.

Responses to Reviewer 2

"So please restructure the paper so that it has clear sections with methods and results for utility and validity, and comment on availability of the questions as well as the platform that you used."
Accepted; we have restructured as requested so that results on validity are made clear in §2.5. The precise elicitation we used is provided in the Supporting Information section. The process itself does not rely on our specific implementation; we have clarified in §3 that the description of our platform is provided as an example.

"If it is a method paper then full data, which demonstrate validity, feasibility, accuracy, and availability need to be made available for reviewers to evaluate them."
Accepted; we have included in §2.5 more details about the accuracy of our validation experiment and a link to our full data from this experiment.

"I believe its is more important to state here they are first reproducible"
Accepted; the term used in §1 Introduction, "reliable", was intended to include computational reproducibility and we have clarified this. A full discussion of issues around computational reproducibility, however, is beyond the scope of this paper.

"make it clear if this is a paper about the platform – or about both."
Accepted; we have clarified in §3 that the online platform is just an example of a practical implementation of the method, which is based on the elicitation procedure plus aggregation methods. We have described elsewhere the technical implementation of the platform so that others could use that, but the method itself could be operationalised in many different ways to suit different contexts.

"A validation method in my view requires use of one or more different methods on the same studies, and showcasing accuracy of your vs the other methods."
Partly accepted; the studies evaluated by participants in the repliCATS project were also the subject of prediction experiments using other techniques. We use the reported results of those experiments for comparison. We have clarified this.

"While I appreciate the challenges section, … . Similarly for future application. These sections are currently too long,"
Accepted; we have reduced the length of these sections.

Responses to Reviewer 3

“this very relevant link that is cited in the paper (https://osf.io/m6gdp/). This is not publicly available and one need to ask for access. It needs to be made publicly available. No restrictions are acceptable.”
Accepted, and with our apologies. We included the private rather than the public link and this error has been fixed. We have confirmed the data availability.

“A reader may have no idea of what the IDEA protocol is”
Accepted; change made as requested. We outline the basic characteristics of the protocol and direct the reader to previously published work that comprehensively describe it.

“In the abstract, again, please elaborate more on the 3 categories "UTILITY, VALIDITY and AVAILABILITY" and detail how each was investigated or addressed.”
Accepted; we emphasise that the “utility of processes to predict replicability is their capacity to test scientific claims without the costs of full replication” and in §3 that this method is available for a range of implementations, not only our implementation.

“Please also add in the abstract the main limitation of the approach to be fair as there are some limitations.”
Accepted; major limitation added to abstract as requested.

“Please elaborate a little bit more on the feasibility of claims like "the IDEA protocol for many experts addressing many problems, with the capacity for the assessment of 3000 claims in 18 months.””
Accepted; more detail provided in §3 about the specific implementation within the SCORE project.

“I do think that results of the pilot study may nicely be illustrated with a figure”
Accepted; figure provided in §2.5 as requested.

“Regarding your answer to the reviewer: "We would happily describe in more detail the advantages and disadvantages of the various standard metrics for prediction outcomes." I agree and support you in working on a figure and or a table to detail this point.”
Accepted; table added in in §2.2 as requested.

“Last panels in Figure 2 are not appropriately ordered and the order should be a, b, c... I understand the restrictions in terms of space, but I really invite you to organise it better.”
Not accepted; table is labelled anticlockwise from centre top. We believe this is a straightforward and readily comprehensible way to organise the information. This has been clarified in the figure caption.
