# Supplementary material for: Predicting reliability through structured expert elicitation with the repliCATS (Collaborative Assessments for Trustworthy Science) process
Source: PLoS One. 2023 Jan 26;18(1):e0274429. doi: 10.1371/journal.pone.0274429 (PMC9879480; doi:10.1371/journal.pone.0274429)
Supplement: Attachment — Submitted filename: Responses_Predicting_reliability_repliCATS.pdf [file pone.0274429.s005.pdf]

Predicting reliability through structured expert elicitation with the repliCATS (Collaborative Assessments for Trustworthy Science) process

In this revision, only Reviewer 2 has requested changes. We accept Reviewer 2's points 1, 3, 5 and partially point 4. We do not accept Reviewer 2's points 2 and 6. Details are provided below.

Responses to Reviewer 2

1) Please rewrite the abstract, to showcase actual data/numbers on Utility, Accuracy, Scalability, Insight and Validity

We accept this recommendation. The abstract has been re-written to include specific figures for Accuracy and Validity, and Scalability. We note that Utility and Insight are qualitative features of the repliCATS process rather than quantitative ones and so specific figures are not included.

2) Please expand your definition of reproducibility in the intro. It is not only about the fact that " multiple observers examining the same data should agree on the facts and the results of analyses" – it is also about the reproducibility of the manuscript itself, and all data/figures produced in it – this aspect requires more attention in the introduction and in the discussion – and its relation to replicability. Additionally, authors should make clear examples of disciplines that replicate their own findings in the primary articles – and therefore do not require replication to be done after study publication. If the culture change would require replications to be included in the publication of a primary study - then the evaluation of strength of claims would be dfferent then they are explained here. Please mention examples of papers and fields that already do this, and why this is not necessary or feasabile for all types of studies. Reproducible manuscript are however feasible.

We do not accept this recommendation. We do not agree with Reviewer 2 that reproducibility is necessarily a prior goal in the assessment of scientific manuscripts, nor is the discussion about practices in different fields in scope for this paper with a well-defined disciplinary range. This recommendation appears to be based on a specific opinion of Reviewer 2, which we do not share, and we do not feel we need to rewrite our manuscript accordingly. We have, however, substantially rewritten the second paragraph of the paper (in lines 9 – 13) to clarify the scope even more explicitly.

3) Please be consistent – you stated in intro: describe the utility, accuracy and scalability

of this method – and yet the section 2 is titled - Utility, Accuracy, Scalability, Insight and Validity. If all will be described, that should be stated.

We accept the recommendation and thank the reviewer for noting the inconsistency in the heading to Section 2. This has been fixed (lines 52-53).

4) I am puzzled again by section 2 - utility should be about the utility of repliCATS project not replication studies themselves. Same applies to the rest. I would advise arranging a call with the editor and agreeing on the structure of this paper.

We partially accept this recommendation. While we believe we had discussed the cost-saving utility of the repliCATS (and similar) processes, we have added additional detail to Section 2.1 in lines (64-66) to make this even more explicit.

5) Section 2.2 did not specify what is the accuracy of repliCATS vs prediction markets, vs surveys – therefore this section also requires rewriting. If as I mentioned in the round 1 review, this paper is about how accuracy will be measured for repliCATS – then this paper needs to be turned into a protocol for all items in section 2.

As discussed, we have restructured Section 2 (lines 67-155) to combine Accuracy and Utility into a single sub-section.

6) I strongly suggest authors create a table where in the rows are Utility, Accuracy, Scalability, Insight and Validity and in 3 columns – surveys, predication markets, and repliCATS project – additional columns can be added – to include actual replication studies or other methods authors believe should be included. And then the paper should be structured in such a way that all of these are covered. Until this is done, I will refrain for making comments on the rest of the manuscript.

We do not accept this recommendation. This would require a complete re-write of the manuscript according to the stylistic preference of Reviewer 2 and we do not consider this to be a reasonable request.
