# Supplementary material for: Predicting reliability through structured expert elicitation with the repliCATS (Collaborative Assessments for Trustworthy Science) process
Source: PLoS One. 2023 Jan 26;18(1):e0274429. doi: 10.1371/journal.pone.0274429 (PMC9879480; doi:10.1371/journal.pone.0274429)
Supplement: Attachment — Submitted filename: PointResponse_PredictingReliability_repliCATS_20120820.docx [file pone.0274429.s003.docx]

Predicting reliability through structured expert elicitation with repliCATS (Collaborative Assessments for Trustworthy Science)

***Original rebuttal letter***

Re: APPEAL_PONE-D-21-05317_20210605

Dear Editors,

All authors appeal against the initial decision to reject our manuscript PONE-D-21-05317 ‘Predicting reliability through structured expert elicitation with repliCATS (Collaborative Assessments for Trustworthy Science)’. This attachment sets out the “point-by-point responses to the reviewers' and/or Academic Editor's comments” as required by the PLOS ONE appeal guidelines (<https://journals.plos.org/plosone/s/editorial-and-peer-review-process#loc-appeals>) Note that the primary reason for this appeal is the failure to assess our manuscript, which was submitted as a Methods paper, against the clear standards for acceptance of such papers, utility and validation, as explicitly described in PLOS ONE submission guidelines. More details about this is contained in our appeal form.

***Point-by-point responses***

*Reviewer’s comments*

*“Nowhere is the claim of accuracy supported by argument, data or even elaborated.”*

As a Methods paper, we are required to provide validation of our method. This was done in §4. We welcome comments that will improve this discussion. We disagree that validation was nowhere supported.

*“As far as I can see the paper consists only of describing their platform and their aspirations. There is no scientific claim to review and nothing for a reviewer to do except to say that this manuscript doesn’t belong in a scientific journal.”*

These reviewer comments suggest that the reviewer is not aware of the fact that PLOS ONE explicitly accepts Methods papers, and is unfamiliar with the guidelines by which such papers should be assessed. §2 describes the background to the field and existing alternatives. §3 describes our method (not our platform).

*“I will say that judging “classification accuracy” by treating “predictions >50% as predictions of replication success and <50% as predictions of replication failure” is extremely maladroit.”*

As described in §2 and throughout, there are multiple measures for prediction accuracy. Classification accuracy is a standard metric within this field, is reported by previous authors, and our use of it allows us to benchmark our method's validity against the existing literature. We would happily describe in more detail the advantages and disadvantages of the various standard metrics for prediction outcomes.

*“Why go to the trouble of eliciting probabilistic predictions?”*

We have described in §3 and throughout the intrinsic advantages of probabilistic estimation, including reducing overconfidence and harnessing diverse viewpoints through mathematical rather than behavioural aggregation. We would happily go into more detail if these arguments were unclear.

*Academic editor’s comments*

The academic editor makes no substantive comment on the manuscript. They say: *“After careful consideration, we have decided that your manuscript does not meet our criteria for publication and must therefore be rejected.”*

As in our primary reason for appeal, we believe that we have met the PLOS ONE “criteria for publication” as a Methods paper.

I look forward to your consideration of this appeal and I expect that you will keep us informed of the progress of this matter.

Regards,

Martin Bush

***Details of revisions***

As described in our rebuttal letter above, the major issue with the original review appears to have been a failure to appreciate our submission as a methods paper. The only substantive question raised was the use of ‘classification accuracy’ which, as described above, is a widely used metric in the field and thus allows meaningful comparisons.

In the absence of substantive comments, we have made no substantive revisions to the manuscript. We have made minor wording changes throughout to emphasize the status of this manuscript as a methods paper. We have also updated a small number of references. Details of these changes can be found in the track changes version.

Regards,

Martin Bush
